# Virus-specific and shared gene expression signatures in immune cells after vaccination in response to influenza and vaccinia stimulation

**DOI:** 10.3389/fimmu.2023.1168784

**Published:** 2023-08-04

**Authors:** Huy Quang Quach, Krista M. Goergen, Diane E. Grill, Iana H. Haralambieva, Inna G. Ovsyannikova, Gregory A. Poland, Richard B. Kennedy

**Affiliations:** ^1^ Mayo Clinic Vaccine Research Group, Division of General Internal Medicine, Mayo Clinic, Rochester, MN, United States; ^2^ Department of Quantitative Health Sciences, Mayo Clinic, Rochester, MN, United States

**Keywords:** influenza vaccine, smallpox vaccine, virus infection, gene expression signature, transcriptomic analysis, pathway analysis

## Abstract

**Background:**

In the vaccine era, individuals receive multiple vaccines in their lifetime. Host gene expression in response to antigenic stimulation is usually virus-specific; however, identifying shared pathways of host response across a wide spectrum of vaccine pathogens can shed light on the molecular mechanisms/components which can be targeted for the development of broad/universal therapeutics and vaccines.

**Method:**

We isolated PBMCs, monocytes, B cells, and CD8^+^ T cells from the peripheral blood of healthy donors, who received both seasonal influenza vaccine (within <1 year) and smallpox vaccine (within 1 - 4 years). Each of the purified cell populations was stimulated with either influenza virus or vaccinia virus. Differentially expressed genes (DEGs) relative to unstimulated controls were identified for each *in vitro* viral infection, as well as for both viral infections (shared DEGs). Pathway enrichment analysis was performed to associate identified DEGs with KEGG/biological pathways.

**Results:**

We identified 2,906, 3,888, 681, and 446 DEGs in PBMCs, monocytes, B cells, and CD8^+^ T cells, respectively, in response to influenza stimulation. Meanwhile, 97, 120, 20, and 10 DEGs were identified as gene signatures in PBMCs, monocytes, B cells, and CD8^+^ T cells, respectively, upon vaccinia stimulation. The majority of DEGs identified in PBMCs were also found in monocytes after either viral stimulation. Of the virus-specific DEGs, 55, 63, and 9 DEGs occurred in common in PBMCs, monocytes, and B cells, respectively, while no DEGs were shared in infected CD8^+^ T cells after influenza and vaccinia. Gene set enrichment analysis demonstrated that these shared DEGs were over-represented in innate signaling pathways, including cytokine-cytokine receptor interaction, viral protein interaction with cytokine and cytokine receptor, Toll-like receptor signaling, RIG-I-like receptor signaling pathways, cytosolic DNA-sensing pathways, and natural killer cell mediated cytotoxicity.

**Conclusion:**

Our results provide insights into virus-host interactions in different immune cells, as well as host defense mechanisms against viral stimulation. Our data also highlights the role of monocytes as a major cell population driving gene expression in *ex vivo* PBMCs in response to viral stimulation. The immune response signaling pathways identified in this study may provide specific targets for the development of novel virus-specific therapeutics and improved vaccines for vaccinia and influenza. Although influenza and vaccinia viruses have been selected in this study as pathogen models, this approach could be applicable to other pathogens.

## 1 Introduction

Although each pathogen has its unique characteristics, pathogen-associated molecular patterns (PAMPs) are conserved across a broad spectrum of pathogens (1). It is well-known that the host relies on limited sets of pattern-recognition receptors (PRRs) to recognize PAMPs, subsequently triggering a variety of signaling pathways as part of the rapid innate immune response (2–4). Therefore, host responses to pathogens are pathogen-specific although certain immune response pathways are shared across multiple pathogens due to the conservation of PAMPs within those pathogens. While understanding pathogen-specific host responses is critical for the development of pathogen-specific therapeutics (5), identifying shared pathways across a wide spectrum of pathogens can shed light on the molecular mechanisms and/or components which can be targeted for the development of broad or even universal therapeutics and vaccines.

The analysis of gene expression signatures in blood leukocytes has been intensively utilized as a robust approach to characterize host responses after infection or vaccination (6). As such, gene transcripts have been used to distinguish a variety of viral infections (7–12) or to differentiate viral from bacterial infections (13–16). This approach has been also applied to study immune responses (17–19) after vaccination. We have recently applied this approach to compare T-cell transcriptional responses to high-dose and adjuvanted influenza vaccines in older adults (20). A common feature in these studies is that gene expression was characterized in peripheral blood mononuclear cells (PBMCs) after a single pathogen stimulation. Therefore, information on shared pathways of host immune responses to multiple vaccine pathogens is limited and subject to confounding due to differences in experimental/analytical approaches between studies.

In the vaccine era, almost everyone receives numerous vaccines in their lifetime, often including 1-2 vaccines annually. We surmised that it would be possible to identify shared biological pathways associated with gene expression across multiple pathogens. We also hypothesized that different cell types of blood leukocytes bear unique gene expression signatures. To test this hypothesis, we analyzed gene expression in PBMCs, monocytes, B cells, and T cells isolated from peripheral blood of donors who previously received both influenza vaccine (within <1 year) and smallpox vaccine (within 1 - 4 years). We then compared and contrasted vaccine-specific gene expression signatures to identify shared pathways associated with transcriptional responses to the two vaccine pathogens across different cell subtypes.

## 2 Materials and methods

The following methods are similar or identical to our previously published studies (20–22).

### 2.1 Ethics statement

This study involved human participants and was approved by The Mayo Clinic Institutional Review Board (IRB# 11-000576). Written informed consents was obtained from each donor before blood collection.

### 2.2 Blood samples and cell separation

Blood samples were obtained from healthy blood donors (n = 10) who received both seasonal influenza vaccine (within <1 year) and smallpox vaccine (within 1 - 4 years). Age and gender characteristics of blood donors are summarized in **Supplemental Table S1**. The peripheral blood mononuclear cells (PBMCs) were isolated from blood, following our previously reported protocol (22). Isolated PBMCs were suspended in a freezing media (20% heat-inactivated FCS, 10% DMSO in RPMI 1640 media) and stored in liquid nitrogen for future use.

MACS MicroBead isolation kits were used to separate monocytes (catalog no. 130-096-537; Miltenyi Biotec, San Diego, CA), B cells (catalog no. 130-091-151), and CD8^+^ T cells (catalog no. 130-096-495) from the PBMCs, following the manufacturer’s protocols. The frequency of monocytes (CD14^+^CD16^+^), B cells (CD19^+^), CD8^+^ T cells (CD3^+^CD8^+^) in the PBMCs and their purities after separation were assessed by flow cytometry.

### 2.3 mRNA sequencing

Cells (1×10^6^) of each population (PBMCs, purified monocytes, B cells, CD8^+^ T cells) were incubated for 6 hours in a 37 °C, 5% CO_2_ incubator under three conditions: i) cell culture media only (RPMI 1640 with glutamine supplemented with 10% FCS, Pen/Strep, nonessential amino acid, HEPES buffer) as unstimulated control, ii) influenza virus (A/H1N1/California/07/2009-like strain) in cell culture media with a virus/cell incubation ratio of 5, and iii) vaccinia virus (New York City Board of Health strain) in cell culture media with the virus/cell incubation ratio of 5. After incubation, the cells were collected and subjected to total RNA extraction using a Qiagen RNeasy Plus Mini Kit (catalog no. 74134) and Qiagen RNAProtect reagent (catalog no. 76104), following a step-by-step instruction in the manufacturer’s protocol. Extracted RNA was qualitatively and quantitatively evaluated by Agilent 2010 Bioanalyzer assay (Agilent, Palo Alto, CA) and a NanoDrop 2000 spectrophotometer (Thermo Fisher Scientific, Waltham, MA).

The extracted RNA (150 ng) was sequenced at Mayo Clinic’s Gene Sequencing Core Facility (Rochester, MN). Briefly, the TruSeq^®^ Stranded mRNA Library Prep v2 (Illumina, San Diego, CA, USA) was used to create cDNA libraries, following the manufacturer’s protocol used at Mayo Clinic Core Facility. Illumina’s HiSeq 2000 S2 Reagent Kit (51 cycles) was used to perform paired-end read on the Illumina HiSeq 2000 Instrument. Gene sequencing data was aligned using the MAP-RSeq V1 pipeline to the h19 human genome as outlined in Kalari et al. (23). The viral sequences were mapped to the vaccinia virus ACAM2000 genome (AY313847.1) and the influenza A/California/07/2009 H1N1 genome.

### 2.4 Statistical analysis

Gene expression was normalized for each cell subset (PBMCs, purified monocytes, B cells, CD8^+^ T cells) using Conditional Quantile Regression (CQN), which adjusts for GC content and gene length bias, via the cqn R package (24). Any post-normalized genes with <16 counts and genes with coefficient of variation in the lower 20^th^ percentile in all groups were removed from further analysis. After filtering, 12,733, 11,641, 12,201, and 12,501 human genes remained for the PBMCs, monocytes, B cells, and T cells, respectively. The edgeR package (25, 26) was used to identify differentially expressed genes (DEG) by fitting quasi-likelihood negative binomial generalized log-linear model, utilizing the offset provided by the CQN normalization and blocking on subject. DEGs were identified based on fold change (FC) of gene expression as compared to corresponding uninfected samples with a threshold set at abs(log2FC) ≥0.585 and *p*-value <0.05. All *p*-values associated with DEG results were unadjusted. Gene set enrichment analysis (GSEA) and over-representation analysis using KEGG pathways was performed using the clusterProfiler package in R (27). Pathway analyses were performed under the settings: i) the minimum geneset size of 3, ii) the maximum geneset size of 800, iii) the *p*-value cutoff of 0.05, and iv) adjusted *p*-values were calculated using the Benjamini-Hochberg method (28). GSEA provides a statistical framework using the hypergeometric distribution to test if KEGG pathways are overrepresented by significant DEGs.

## 3 Results

### 3.1 Differentially expressed genes in influenza-stimulated cells

**Figure 1:**
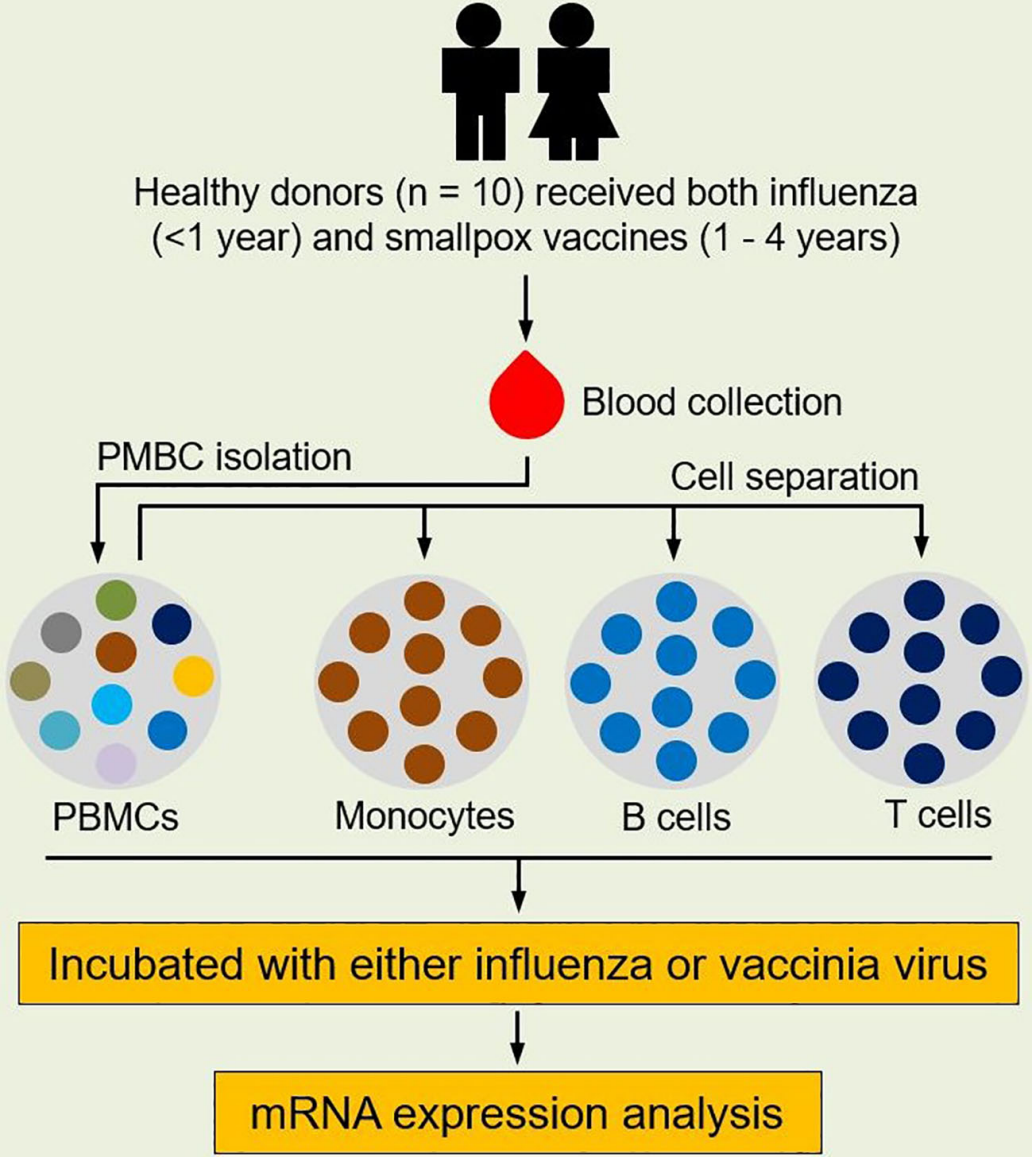
Study design. Healthy subjects (n = 10) who received both seasonal influenza vaccine (within the prior year) and smallpox vaccine (with the prior 1-4 years) were enrolled for this study. Blood was sampled from each subject and PBMCs were isolated from blood. Monocytes, B cells, and CD8^+^ T cells were separated from PBMCs using MACS MicroBead isolation kits. Each cell population was then incubated with either influenza virus (A/H1N1/California/07/2009-like strain) or vaccinia virus (NYCBOH strain). Cells in cell culture media served as unstimulated controls. After infection, RNA was extracted from incubated cells and gene expression in each cell population was profiled.

**Figure 2:**
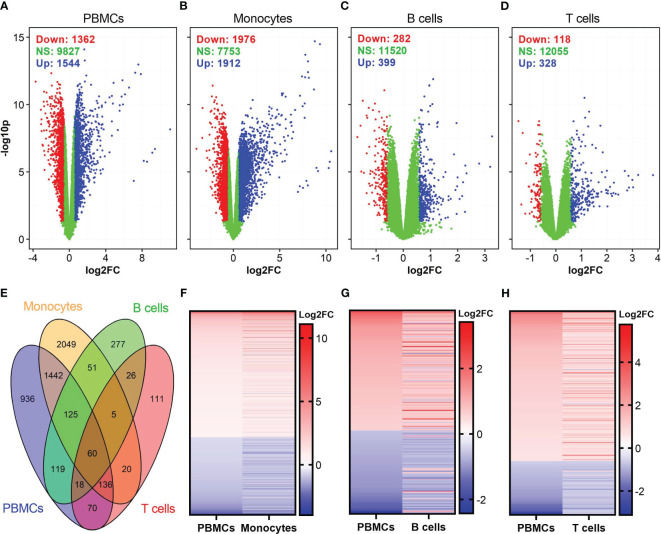
Identification of differentially expressed genes (DEGs) in influenza-stimulated cells. **(A–D)** Volcano plots show the distributions of DEGs in PBMCs, monocytes, B cells, and CD8^+^ T cells. DEGs are defined as genes with *p <*0.05 and fold change (FC) >1.5, equivalent to log2FC >0.585 (for up-regulated genes) or <0.67 (equivalent to log2FC <-0.585 for down-regulated genes) as compared to corresponding unstimulated controls. Down, down-regulated; NS, nonsignificantly different; Up, up-regulated genes. **(E)** Venn diagram shows overlapped DEGs among PBMCs, monocytes, B cells, and CD8^+^ T cells. **(F–H)** A comparison of expression pattern of overlapped DEGs between PBMCs and monocytes **(F)**, PBMCs and B cells **(G)**, PBMCs and CD8^+^ T cells **(H)**. For comparison purpose in **(F–H)** the levels of gene expression in PBMCs were sorted.

After blood collection, we isolated PBMCs, and purified monocytes, B cells, and CD8^+^ T cells were using commercial magnet-based separation kits (**Figure 1**). We incubated each of these cell populations with either influenza virus or vaccinia virus and profiled gene expression in each cell subset (**Figure 1**). Differential gene expression analysis identified a total of 2,906, 3,888, 681, and 446 differentially expressed genes (DEGs) in influenza-stimulated PBMCs, monocytes, B cells, and CD8^+^ T cells, respectively (**Figures 2A–D**). Interestingly, PBMCs and monocytes shared 1,763 DEGs, while PBMCs had 322 and 284 DEGs in common with B cells and CD8^+^ T cells, respectively (**Figure 2E**). In overlapping DEGs, we observed a similar pattern of gene expression in PBMCs and monocytes (**Figure 2F**). The expression patterns of DEGs were also similar between PBMCs and B cells (**Figure 2G**), and between PBMCs and CD8^+^ T cells after influenza stimulation (**Figure 2H**), although some genes differentially regulated in B cells and CD8^+^ T cells.

Among the DEGs in each cell population, the highest fold changes of gene expression were observed in PBMCs and monocytes (**Table 1**). PBMCs and monocytes also shared numerous DEGs, including seven of top ten up-regulated genes (IFNA1, IFNA2, IFNA7, IFNA10, IFNA13, IFN17, TEKT1) and two of top ten down-regulated genes (CCL24 and SCRT2) (**Table 1**). In contrast, the expression level of DEGs in B cells and T cells were ~100-fold lower than that in PBMCs and monocytes (**Table 1**). Both B cells and T cells did not share any DEGs with PBMCs among their top DEGs (**Table 1**). Meanwhile, B cells and T cells shared some genes encoding for interferon induced proteins, such as IFIT1, RSAD2, among their top DEGs (**Table 1**).

**Table 1:** Top DEGs in influenza-stimulated cells.

Top 20 DEGs in influenza-stimulated PBMCs							
No.	Gene name	Fold change	*p* value	No.	Gene name	Fold change	*p* value
1	IFNA8	2181.955	7.04E-09	11	FNDC4	0.145373	8.44E-09
2	IFNA14	726.0143	1.73E-16	12	RASAL1	0.145041	1.1E-11
3	IFNA1	678.3852	1.98E-07	13	C5orf20	0.141976	6.08E-11
4	IFNA2	607.6028	3.85E-17	14	SCRT2	0.133294	1.14E-07
5	IFNA7	559.336	3.74E-07	15	NRG1	0.128385	9.47E-08
6	IFNA17	373.914	1.76E-06	16	STEAP4	0.119966	9.26E-09
7	IFNA13	291.8295	1.52E-06	17	CXCL6	0.119082	1.26E-09
8	SERPINB10	232.9731	5.44E-13	18	ALOX15B	0.116041	2.87E-09
9	TEKT1	193.4793	1.06E-13	19	MYCL1	0.11461	2.4E-10
10	IFNA10	151.9787	4.34E-13	20	CCL24	0.076289	1.98E-12
Top 20 DEGs in influenza-stimulated monocytes							
1	IFNA1	1511.601	2.92E-07	11	SHF	0.172435	2.15E-09
2	IFNA7	1362.34	1.78E-06	12	DTX1	0.167912	1.3E-06
3	IFNA17	1263.123	5.21E-06	13	EMILIN1	0.167355	1.72E-08
4	IFNA5	744.4645	2.71E-16	14	LMCD1	0.165757	2.45E-07
5	IFNA16	656.0336	3.28E-15	15	SYT1	0.141535	9.13E-07
6	MS4A5	568.8181	8.83E-06	16	C21orf67	0.135241	2.74E-07
7	IFNA13	505.4156	4.98E-06	17	CMTM8	0.131482	8.34E-06
8	IFNA2	447.5793	4.8E-16	18	CCL24	0.09982	1.8E-10
9	TEKT1	445.7084	1.98E-15	19	OLFM2	0.097763	3.03E-08
10	IFNA10	417.3111	7.5E-12	20	SCRT2	0.063292	1.95E-07
Top 20 DEGs in influenza-stimulated B cells							
1	OASL	9.618311	2.31E-08	11	SCD	0.414793	1.75E-10
2	IFIT1	9.153269	4.22E-06	12	PEG10	0.399879	9.61E-10
3	CMPK2	6.92906	2.39E-06	13	TUBB4A	0.394574	4.17E-07
4	IFIT3	5.018015	1.28E-05	14	C3orf37	0.393327	6.55E-10
5	IFI44L	4.864317	3.43E-06	15	C1orf233	0.393102	0.00038
6	CCL2	4.823913	0.00922	16	KLF15	0.367394	1.2E-05
7	RSAD2	4.599041	5.89E-05	17	SLC38A11	0.356771	5.22E-11
8	BCL2L14	4.491828	0.000623	18	NPW	0.344144	9.47E-06
9	OLR1	4.210285	0.001037	19	LOC100507254	0.337172	3.71E-09
10	IFI44	3.98739	2.55E-05	20	CTGF	0.311241	2.57E-08
Top 20 DEGs in influenza-stimulated T cells							
1	IFIT1	13.98099	1.76E-05	11	GDF10	0.511657	0.001692
2	CXCL11	9.346556	1.7E-05	12	PRSS23	0.493313	5.07E-07
3	RSAD2	8.150847	0.000141	13	MIR4323	0.489808	0.012527
4	IFI44L	7.970153	3.95E-05	14	PODN	0.483733	0.000127
5	CMPK2	7.262956	0.000144	15	ADAMTS1	0.476493	0.000123
6	CXCL10	7.177282	1.95E-05	16	CMKLR1	0.446075	2.32E-06
7	IFIT3	6.931951	0.00024	17	MYCL1	0.435182	0.012756
8	CXCL9	6.493048	8.53E-06	18	ITGAM	0.405805	1.29E-08
9	USP18	5.441969	0.000483	19	CXCR1	0.391311	0.002656
10	IFI6	5.069574	0.000428	20	CX3CR1	0.360917	1.67E-09

In each cell subset, top 10 up-regulated genes (No. 1-10) and top 10 down-regulated genes (No. 11-20) were selected based on their fold changes of expression compared to corresponding unstimulated controls. Full list of DEGs and their additional information are available in **Supplemental Tables S2–S5**.

**Figure 3:**
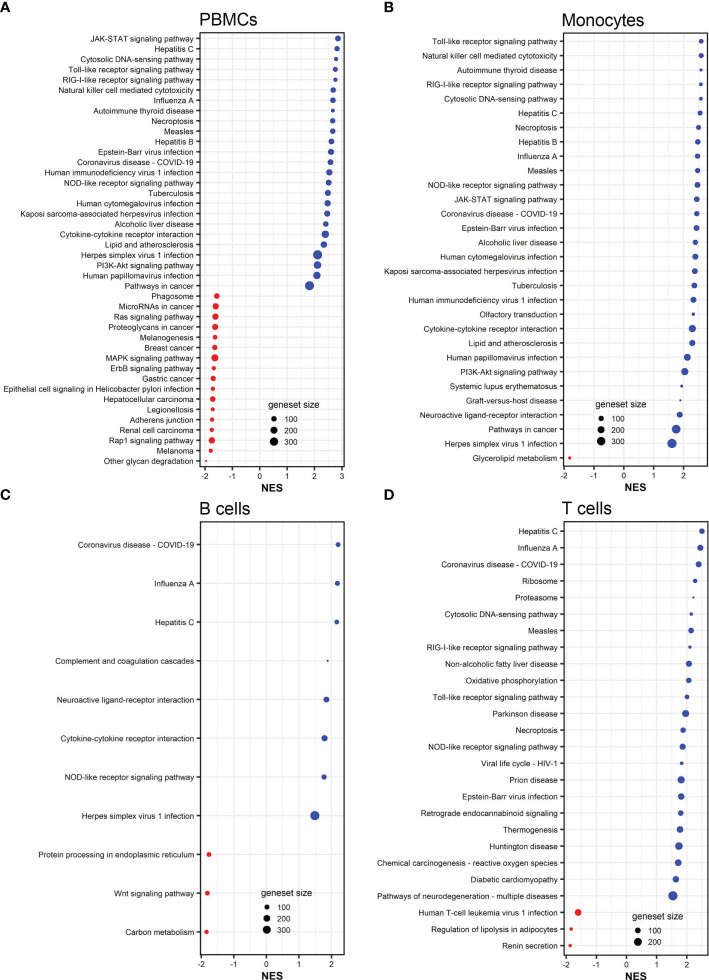
KEGG pathways associated to DEGs in influenza-stimulated PBMCs **(A)**, monocytes **(B)**, B cells **(C)**, and CD8^+^ T cells **(D)**. All KEGG pathways shown are statistically significant (adjusted *p <*0.05). NES, normalized enrichment score. Red color (NES <0) indicates suppressed pathways while bule color (NES >0) indicates activated pathways. The size of each red or blue dot represents the total number of genes (size of geneset) in the pathways. Additional information of these pathways is provided in **Supplemental Tables S10–S13**.

To further understand the biological pathways associated with the DEGs, we performed gene set/pathway enrichment analysis using the clusterProfiler package and KEGG modules and pathways (27). We found that DEGs in PBMCs, monocytes, B cells, and CD8^+^ T cells were significantly enriched in 42, 30, 11, and 26 pathways (adjusted *p <*0.05), respectively (**Figure 3**). Influenza A and coronavirus disease-COVID-19 pathways were among the top enriched pathways in all influenza-stimulated cell populations (**Figure 3**). The NOD-like receptor signaling pathway was also enriched in each of the four cell populations upon influenza stimulation (**Figure 3**). Similar to the pattern of gene expression (**Figure 2F**), almost all signaling pathways associated with DEGs in monocytes were also found in PBMCs, including JAK-STAT signaling pathway, cytosolic DNA-sensing pathway, Toll-like receptor signaling pathway, RIG-I-like signaling pathway, cytokine-cytokine receptor pathway, and PI3K-Akt signaling pathway (**Figure 3**).

### 3.2 Differentially expressed genes in vaccinia-stimulated cells

**Figure 4:**
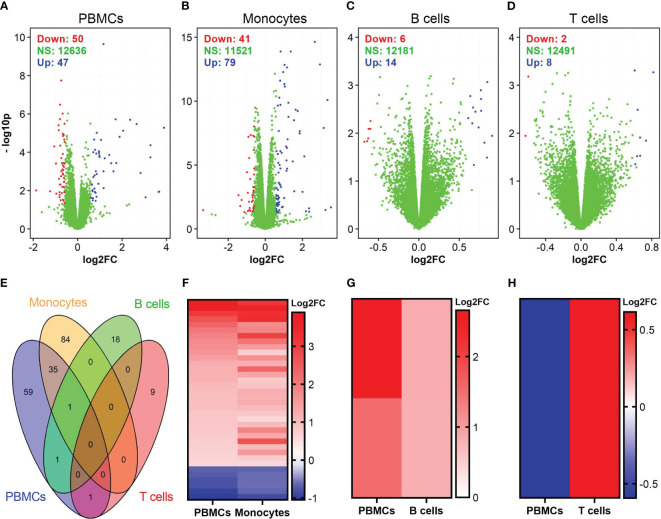
Identification of DEGs in vaccinia-stimulated cells. **(A–D)** Volcano plots show the distributions of DEGs in PBMCs, monocytes, B cells, and CD8^+^ T cells incubated with vaccinia virus. **(E)** Venn diagram shows overlapped DEGs among vaccinia-stimulated PBMCs, monocytes, B cells, and CD8^+^ T cells. **(F–H)** A comparison of expression pattern of overlapped DEGs between PBMCs and monocytes **(F)**, PBMCs and B cells **(G)**, PBMCs and CD8^+^ T cells **(H)**. For comparison purpose in **(F–H)**, the levels of gene expression in PBMCs were sorted.

We also identified DEGs in vaccinia-stimulated cells (**Figure 4**). In general, the number of DEGs in vaccinia-stimulated cells was approximately 30-fold less than that in the influenza-stimulated cells. Specifically, a total of 97, 120, 20, and 10 DEGs were identified in PBMCs, monocytes, B cells, and CD8^+^ T cells after vaccinia stimulation, respectively (**Figures 4A–D**). Of vaccinia-induced DEGs, 36 DEGs were identified in both PBMCs and monocytes (**Figure 4E**). We also observed a similar expression pattern of overlapping DEGs in PBMCs and monocytes (**Figure 4F**), PBMCs and B cells (**Figure 4G**). Only one common DEG was identified in PBMCs and CD8^+^ T cells, but its expression was in opposite directions (**Figure 4H**). Similar to influenza stimulation (**Table 1**), vaccinia-stimulated PBMCs and monocytes shared multiple interferon-encoding genes, such as IFNA7, IFNA17, among their top DEGs (**Table 2**).

**Table 2:** Top DEGs in vaccinia-stimulated cells.

Top 20 DEGs in vaccinia-stimulated PBMCs							
No.	Gene name	Fold change	*p* value	No.	Gene name	Fold change	*p* value
1	HIST1H4B	14.82992	5.3E-06	11	SIGLEC15	0.56646	0.012112
2	IFNA17	12.72248	0.01125	12	OR2B11	0.56639	0.02211
3	IFNA8	12.50059	0.011594	13	XIRP1	0.55963	0.002415
4	IFNA2	9.718248	4.3E-05	14	CCL22	0.55423	7.1E-06
5	IFNA14	9.684393	0.000185	15	ZNF366	0.550802	3.89E-06
6	IFNA7	8.327709	0.022589	16	CCL8	0.514673	0.014367
7	IFNA10	7.110967	0.001202	17	CLEC4F	0.507814	0.000387
8	HIST1H4E	6.306492	3.37E-06	18	GREM1	0.48265	4.95E-05
9	VTRNA1-3	5.144499	2.02E-06	19	OR9A4	0.423488	0.010843
10	HIST1H4C	4.131405	7.48E-06	20	CCL11	0.27434	0.009736
Top 20 DEGs in vaccinia-stimulated monocytes							
1	IFNA17	11.63274	0.020463	11	HYAL4	0.547074	0.007491
2	HIST1H4E	10.21971	7.98E-11	12	NRCAM	0.532147	6.28E-08
3	IFNA7	9.452872	0.032506	13	C10orf81	0.523123	0.039039
4	HIST1H4B	8.827368	1.25E-08	14	SNORA16B	0.522103	0.001467
5	HSPA4L	7.710156	1.34E-13	15	MTUS1	0.506773	3.63E-05
6	SERPINH1	6.382744	2.3E-15	16	LINC00487	0.460149	0.048456
7	MRVI1-AS1	5.148515	0.045137	17	AMH	0.412942	0.005379
8	IFNA5	5.134458	0.024392	18	FOXD4	0.392176	0.015616
9	ESPNL	4.556629	0.041009	19	EPHA6	0.368833	0.002251
10	CYP7A1	4.295212	2.08E-06	20	METTL21C	0.098127	0.03412
Top 16 DEGs (10 up-regulated and 6 down-regulated genes) in vaccinia-stimulated B cells							
1	MIR374B	1.876026	0.011394	9	LOC100133985	1.62534	0.003572
2	HIST1H4E	1.800007	0.00086	10	MTRNR2L7	1.603618	0.046264
3	MIR3679	1.793666	0.03245	11	KLF15	0.661182	0.005598
4	HIST1H1D	1.754327	0.015881	12	RAX2	0.660299	0.008124
5	SCARNA1	1.706011	0.003422	13	PLEKHA4	0.64964	0.008154
6	IQGAP3	1.705527	0.001947	14	SLC35G5	0.647834	0.012933
7	LOC91450	1.702022	0.001277	15	MYLPF	0.642104	0.01482
8	TAS2R46	1.669156	0.007607	16	ANP32AP1	0.626549	0.01517
Top 10 DEGs (8 up-regulated and 2 down-regulated genes) in vaccinia-stimulated T cells							
1	MIR4467	1.764117	0.000538	6	LOC100303749	1.553224	0.030204
2	RNF223	1.663955	0.014388	7	UQCRBP1	1.530758	0.044532
3	MTRNR2L6	1.590673	0.029816	8	SRRM4	1.523065	0.000494
4	MIR101-1	1.58819	0.012361	9	SRGAP3	0.662062	0.000663
5	HCG9	1.55889	0.003262	10	KLF1	0.647852	0.011399

In vaccinia-stimulated PBMCs and monocytes, top 10 up-regulated genes (No. 1-10) and top 10 down-regulated genes (No. 11-20) were selected based on their fold changes of expression compared to corresponding unstimulated controls. In vaccinia-stimulated B cells, top 10 up-regulated genes (No. 1-10) were also selected based on their fold changes, but only 6 DEGs (No. 11-16) were down-regulated. Similarly, in vaccinia-stimulated CD8^+^ T cells, 8 (No. 1-8) and 2 DEGs (No. 9-10) were up-regulated and down-regulated, respectively. Full list of DEGs and their additional information are available in **Supplemental Tables S6–S9**.

**Figure 5:**
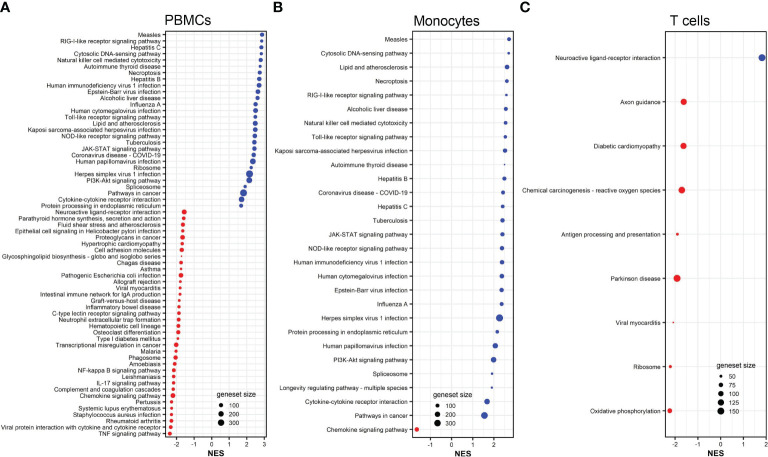
KEGG pathways associated to DEGs in vaccinia-stimulated PBMCs **(A)**, monocytes **(B)**, and CD8^+^ T cells **(C)**. All KEGG pathways shown are statistically significant (adjusted *p <*0.05). NES, normalized enrichment score. Red color indicates suppressed pathways (NES <0) while bule color indicates activated pathways (NES >0). The size of each red or blue dot represents the total number of genes (size of geneset) in the pathways. Additional information of these pathways is provided in **Supplemental Tables S14–S16**.

Gene set enrichment analysis demonstrated that vaccinia-induced DEGs in PBMCs, monocytes, and CD8^+^ T cells were significantly (adjusted *p <*0.05) enriched in 64, 29, and 9 pathways (**Figure 5**). No pathway was significantly associated with vaccinia-induced DEGs in B cells. Similar to influenza-induced DEGs (**Figures 3A, B**), vaccinia-induced DEGs in PBMCs and monocytes were enriched in multiple innate pathways, including cytosolic DNA-sensing pathway, RIG-I receptor signaling pathway, Toll-like receptor signaling pathway, JAK-STAT signaling pathway, and NOD-like receptor signaling pathway (**Figures 5A, B**).

### 3.3 Shared DEGs in each cell population after influenza and vaccinia stimulation

**Figure 6:**
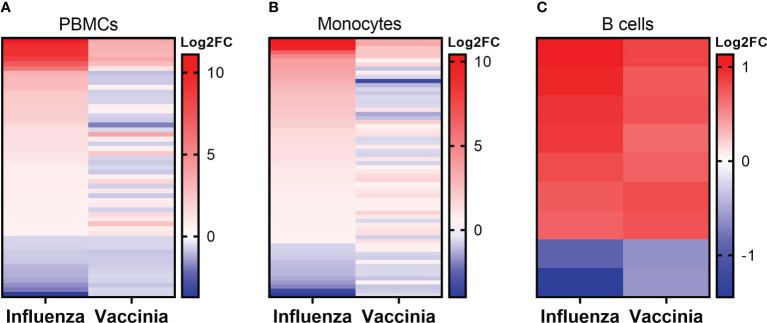
Expression patterns of overlapped DEGs in PBMCs **(A)**, monocytes **(B)**, and B cells **(C)** after influenza and vaccinia stimulation. No overlapped DEGs were identified in CD8+ T cells. For comparison purpose, the expression levels of DEGs in influenza-stimulated cells were sorted.

We next explored the commonalities of DEGs in each cell population after influenza and vaccinia stimulation. Of DEGs induced either by influenza or vaccinia virus, we identified a total of 55, 63, and 9 shared DEGs in PBMCs, monocytes, and B cells, respectively (**Figure 6**). Meanwhile, no shared DEGs were identified in CD8^+^ T cells. Although we observed a similar expression pattern in PBMCs and monocytes after viral stimulation; a significant number of shared DEGs were expressed in different directions upon influenza and vaccinia stimulation (**Figures 6A, B**). In contrast, shared DEGs in B cells were expressed similarly after influenza and vaccinia stimulation (**Figure 6C**).

**Figure 7:**
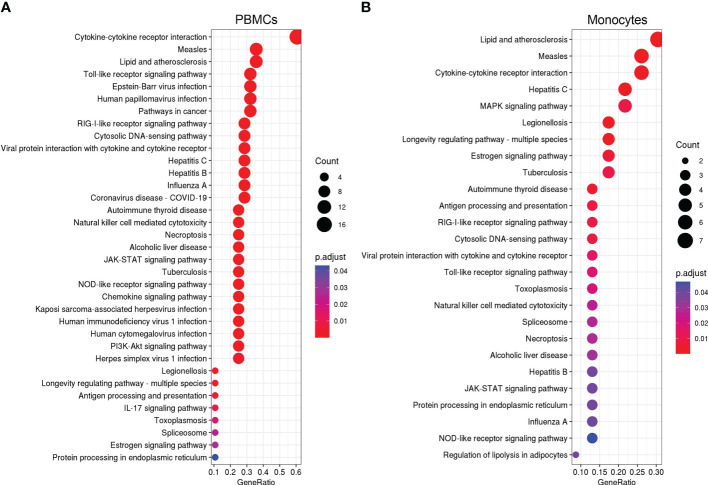
KEGG over-representation pathways associated to overlapped DEGs in PBMCs **(A)** and monocytes **(B)** after influenza and vaccinia stimulation. All KEGG over-presentation pathways shown are statistically significant (adjusted *p <*0.05). The size of each dot represents the count of DEGs in associated pathways. The adjusted *p* value (p.adjust as shown in the figures) is color coded. Additional information of these pathways is provided in **Supplemental Tables S17–S19**.

We further identified over-represented pathways associated with the shared DEGs after influenza and vaccinia stimulation to understand their biological functions. We found that shared DEGs in PBMCs and monocytes were significantly over-represented in 35 and 26 biological pathways, respectively (**Figure 7**). These pathways included: cytokine-cytokine receptor interaction, Toll-like receptor signaling pathway, NOD-like receptor signaling pathway, RIG-I-like receptor signaling pathway, viral protein interaction with cytokine and cytokine receptor, JAK-STAT signaling pathways, cytosolic DNA-sensing pathway, and natural killer cell mediated cytotoxicity. In contrast, only one shared DEG was identified in B cells and it was significantly associated with the taste transduction pathway.

## 4 Discussion

Understanding host transcriptomic responses to pathogens after vaccination is critical for the development of vaccines (5). In this study, we identified virus-specific DEGs in PBMCs, monocytes, B cells, and T cells isolated from healthy donors who had been immunized with both seasonal influenza vaccine and smallpox vaccine. Among the virus-specific DEGs, we were able to identify shared DEGs and their associated biological pathways after influenza and vaccinia virus stimulation. Our data showed a significant number of innate signaling pathways associated with shared DEGs were activated in response to influenza and vaccinia virus stimulation. Our data also highlighted the role of monocytes as a cell population driving transcriptomic responses in PBMCs to both viruses. These results suggest that this specific cell type may be useful as sentinels to monitor the physiologic response during an infection or after vaccination.

We chose influenza and smallpox vaccines as vaccine models for multiple reasons. First, influenza vaccine is administered annually in the U.S.; therefore, it is a logical choice to study. Meanwhile, after its eradication in 1980, routine smallpox vaccination ceased (29). However, vaccinia virus is used as a vector for the development of multiple vaccines. Public concern still exists concerning the safety of these vaccines and there have been an increased number of zoonotic orthopoxvirus outbreaks. A recent global re-emergence of monkeypox (30), another member of *Poxviridae*, further heightens this public concern. As a result, vaccinia virus serves as a re-emerging pathogen model which may require a vaccination campaign to re-establish herd immunity (31, 32). Second, by choosing two distantly related viruses, such as influenza (a small RNA virus that replicates in the nucleus) and vaccinia (a large DNA virus that replicates in the cytoplasm), we aimed to demonstrate that shared signaling pathways of host responses exist across highly diverse pathogens. In other words, we demonstrated that some shared signaling pathways were activated in response to the stimulation of a wide spectrum of pathogens. Therefore, these signaling pathways should be principal pathways in response to viral infections and identifying these pathways will aid the development of general or even universal therapeutics and vaccines.

Previously, we profiled transcriptomic responses in blood leukocytes after influenza vaccination (21, 33–37) and smallpox vaccination (38–42). We and others repeatedly observed the activation of interferon-encoding genes in PBMCs after either influenza (35–37, 43) or vaccinia stimulation (38, 40). Consistent to these previous findings, in this study we found that interferon-encoding genes were up-regulated in both PBMCs and monocytes stimulated with either influenza virus (**Table 1**) or vaccinia virus (**Table 2**). Transcriptional analysis has also found that interferon-encoding genes were among the top activated genes in human PBMCs infected with different viruses (7, 12). Together, these findings consolidate a defensive role of interferons in viral infections. Serving as the first line of innate defense, interferon production is amongst the first innate responses against viral infection (44, 45); hence, this could explain for the up-regulation of interferon-encoding genes observed in this and other studies. In turn, the production of interferons is activated by the JAK-STAT signaling pathway (44–46), which was consistently amongst the top enriched signaling pathways in this study (**Figures 3**, **5**, **7**). Altogether, these findings suggest the JAK-STAT signaling pathway as a main target for the development of viral therapeutics or vaccines (46).

After influenza stimulation, the identified DEGs were enriched in influenza A and coronavirus disease-COVID-19 pathways in each cell population (**Figure 3**). These results suggest a similarity between these two respiratory viruses in the way they are recognized and targeted by the host immune system, as observed by others across respiratory viruses (9, 47). More importantly, even with the influenza and vaccinia viruses used in this study, we could identify 35 and 26 biological pathways associated with shared DEGs in PBMCs and in monocytes, respectively (**Figure 7**). These shared pathways were mainly involved in the activation of innate immune responses, such as NOD-like, Toll-like, RIG-I-like receptor signaling pathways, cytokine-cytokine receptor interaction, cytosolic DNA-sensing pathway, and natural killer cell mediated cytotoxicity (**Figure 7**). Altogether, these observations suggest that the host relies on a similar set of PRRs to sense the presence of pathogens and trigger similar innate immune pathways in responses to pathogens (1–4, 48). Therefore, these innate immune pathways could potentially be targeted for the development of broader viral therapeutics or be used to inform the use of appropriate adjuvants for vaccines.

Consistently, we observed the similarity in DEGs and their expression patterns in PBMCs and in monocytes upon either influenza or vaccinia (**Tables 1**, **2**; **Figures 2A, B, F**, **4A, B, F**, **6A, B**). As a result, the biological pathways associated with the DEGs in PBMCs and in monocytes were also similar (**Figures 3A, B**, **5A, B**, **7A, B**). These results reflect the importance of monocytes as a cell population driving transcriptomic responses. This may be due to the remarkable degree of plasticity observed in myocytes, in which they can rapidly sense and respond to diverse invaders (49, 50). Our results suggest that monocytes could be a major target cell type for viral therapeutics and/or serve as a cellular biomarker for infection/vaccination. In fact, previous studies have found that monocytes are the main target for a variety of viral infections, including influenza, dengue, measles, Zika (51–54). Our results further demonstrate the heterogenous nature of PBMC samples and the limitations in using whole blood or PBMCs to evaluate transcriptomic changes after infection or vaccination. While useful as an initial step, examination of isolated cell subsets or the use of single cell technologies are more powerful and increasingly taking the place of bulk RNA-Seq.

In contrast, B cells and CD8^+^ T cells provided limited information on transcriptomic responses and the biological pathways associated with DEGs in B cells and CD8^+^ T cells did not show a specific trend (**Figures 2**–**5**). This could be due to the fact that receptors on B cells and T cells are binding to specific viral epitopes, rather than DAMPs. B cells and T cells are involved in adaptive immune responses, which chronologically occurs after the activation of innate responses triggered by PRRs.

Our study has both strengths and limitations. The strengths of this study mainly lie in our study design. As such, first we studied the transcriptomic responses induced by two viral vaccine pathogens, which minimizes variation and confounding due to the differences in experimental/analytical approaches between studies. To the best of our knowledge, this study is one of the first studies identifying shared transcriptomic responses induced by these two vaccine pathogens in the same host. Second, rather than study only PBMCs we expanded the transcriptomic characterizations in purified monocytes, B cells, and T cells. By studying the transcriptomic responses in these purified cell subset populations and comparing the responses in these cell populations with the responses in PBMCs, we found that the transcriptomic responses in PBMCs were driven by monocytes. Meanwhile, our observations were limited to 10 subjects, mainly due to the difficulty in recruiting study participants who met the requirements for this study. A future study with the same design, but with a larger study cohort may consolidate the results observed in this study. In this study, we used only two virus models. A future study including more pathogens of different types, such as bacteria, virus, fungi would facilitate the development of universal therapeutics or vaccines for a broader spectrum of pathogens. In addition, we used fold change of gene expression and unadjusted *p* value to identify DEGs. While the fold change shows a relative expression of each individual gene in virus-infected cells as compared to control cells, unadjusted *p* value may be impacted by multiple testing, where the expression of a certain gene is influenced by others (55).

In conclusion, we identified both virus-specific and shared DEGs in response to influenza and vaccinia stimulation and their associated biological pathways in this study. Our results highlight monocytes as a main cell type driving transcriptomic responses in the PBMCs. The shared biological signaling pathways observed in this study may provide molecular insights into virus-host interactions, serving as targets for the development of novel virus-specific therapeutics and new generic vaccines for vaccinia and influenza. Although influenza and vaccinia viruses have been selected in this study as pathogen models, we believe that this approach is applicable to other pathogens including SARS-CoV-2.

## Data availability statement

The data presented in the study are deposited in Synapse, under Project SynID syn52165181.

## Ethics statement

This study was approved by The Mayo Clinic Institutional Review Board. Each study participant provided their written informed consents before blood collection.

## Author contributions

HQ analyzed data, wrote, and revised the manuscript. KG and DG analyzed mRNA sequencing data and reviewed the manuscript. RK, IH, and IO carried out the experiments, and critically reviewed and edited the manuscript. GP and RK acquired research funding, supervised the project, and edited the manuscript. All authors contributed to the article and approved the submitted version.
